# Sex differences in sympathetic innervation and browning of white adipose tissue of mice

**DOI:** 10.1186/s13293-016-0121-7

**Published:** 2016-12-09

**Authors:** Sang-Nam Kim, Young-Suk Jung, Hyun-Jung Kwon, Je Kyung Seong, James G. Granneman, Yun-Hee Lee

**Affiliations:** 1 College of Pharmacy, Yonsei University, 310 Veritas Hall D, 85 Songdogwahak-ro, Yeonsu-gu Incheon, 21983 South Korea; 2 College of Pharmacy, Pusan National University, Busan, 46241 South Korea; 3 BK21 PLUS Program for Creative Veterinary Science Research, Research Institute for Veterinary Science, and Korea Mouse Phenotyping Center, Seoul National University, Seoul, 08826 South Korea; 4 School of Medicine, Wayne State University, Detroit, MI 48201 USA

**Keywords:** Brown adipocytes, UCP1, Sympathetic innervation, White adipose tissue, Sex differences

## Abstract

**Background:**

The higher prevalence of obesity-related metabolic disease in males suggests that female sex hormones provide protective mechanisms against the pathogenesis of metabolic syndrome. Because browning of white adipose tissue (WAT) is protective against obesity-related metabolic disease, we examined sex differences in β3-adrenergic remodeling of WAT in mice.

**Methods:**

Effects of the β3-adrenergic receptor agonist CL316,243 (CL) on browning of white adipose tissue were investigated in male and female C57BL mice. The role of ovarian hormones in female-specific browning was studied in control female C57BL mice and mice with ovarian failure induced by 4-vinylcyclohexene diepoxide treatment for 15 days.

**Results:**

We found that treatment with CL-induced upregulation of brown adipocyte markers and mitochondrial respiratory chain proteins in gonadal WAT (gWAT) of female mice, but was without effect in males. In contrast, CL treatment was equally effective in males and females in inducing brown adipocyte phenotypes in inguinal WAT. The tissue- and sex-specific differences in brown adipocyte recruitment were correlated with differences in sympathetic innervation, as determined by tyrosine hydroxylase immunostaining and western blotting. Levels of the neurotrophins NGF and BDNF were significantly higher in gWAT of female mice. CL treatment significantly increased NGF levels in gWAT of female mice but did not affect BDNF expression. In contrast, estradiol treatment doubled BDNF expression in female adipocytes differentiated in vitro. Ovarian failure induced by 4-vinylcyclohexene diepoxide treatment dramatically reduced BDNF and TH expression in gWAT, eliminated induction of UCP1 by CL, and reduced tissue metabolic rate.

**Conclusions:**

Collectively, these data demonstrate that female mice are more responsive than males to the recruitment of brown adipocytes in gonadal WAT and this difference corresponds to greater levels of estrogen-dependent sympathetic innervation.

**Electronic supplementary material:**

The online version of this article (doi:10.1186/s13293-016-0121-7) contains supplementary material, which is available to authorized users.

## Background

Increased adiposity positively correlates with higher susceptibility to metabolic disease, yet this correlation is modified by sex [1, 2]. The greater prevalence of obesity-related metabolic disease in males suggests that female sex hormones provide protective mechanisms against the pathogenesis of metabolic syndrome, possibly by modulating metabolic phenotypes in adipose tissue.

Adipose tissue can store excess energy, mainly as triglyceride, and mobilize free fatty acids (FFA) in response to systemic needs, thereby contributing to energy homeostasis [3]. Dysregulation of lipid metabolism in adipose tissue can lead to ectopic lipid accumulation in non-adipose organs. This results in lipotoxicity, which is a major player in the development of insulin resistance and obesity-related metabolic disease [1, 4].

In general, adipose tissue can be subcategorized into white and brown adipose tissue [1]. A main physiological role of white adipose tissue (WAT) is to buffer fluctuating energy supply, while brown adipose tissue (BAT) is specialized for non-shivering thermogenesis to maintain body temperature [5]. In brown adipocytes, uncoupling protein 1 (UCP1) can uncouple the mitochondrial proton gradient from ATP synthesis during oxidative phosphorylation to generate heat [5]. Thus, high levels of mitochondria and UCP1 expression specify the metabolic phenotype of brown adipocytes [5]. In addition to constitutive brown adipocytes in classic brown adipose tissue depots [5], brown adipocytes can appear in WAT depots in response to cold temperature and β-adrenergic stimulation [6, 7]. Inducible brown adipocytes in WAT are considered a distinct cell type, and called beige (or BRITE, for BRown in whITE) adipocytes [8, 9]. Non-shivering thermogenesis in brown and beige/BRITE adipocytes has been studied as a means to increase energy expenditure and therefore as a potential therapeutic target to treat metabolic disease associated with obesity [3].

The ability to recruit brown adipocytes in WAT varies depending on the anatomical location of the adipose tissue depots [1, 7]. The reasons for this variation are not clear but could involve distinct committed lineages or extrinsic factors, like tissue microenvironment. Microenvironmental factors that can affect adipose tissue function include vascularization, variation in local growth factors, and peripheral sympathetic innervation [1]. While BAT is more densely innervated by peripheral sympathetic nerves than WAT [10, 11], innervation levels of adipose tissues positively correlate with the ability to recruit brown adipocytes in WAT [12]. For example, subcutaneous inguinal white adipose has greater sympathetic innervation and higher norepinephrine turnover rate [11, 13] compared to gonadal fat depots. Although activation of brown/beige adipocytes by sympathetic nerve activity improves metabolic profiles [14, 15], the factors that control physiological sympathetic innervation levels in adipose tissue from various anatomic locations are not fully understood.

Because females are more resistant to obesity-related metabolic disease, sex hormones have been suggested as major factors leading to sexual dimorphism in the pathogenesis of metabolic diseases [16]. Indeed, crucial roles of female sex hormones in adipose tissue metabolism have been demonstrated [16, 17], and it has been shown that estrogen can directly activate lipolysis through estrogen receptor alpha signaling in adipocytes [17–19]. In addition to direct activation of lipolysis, sex hormones influence body fat distribution, and subcutaneous fat is more abundant in women [2]. However, sex differences in sympathetic innervation and the induction of thermogenic adipocytes in anatomically analogous WAT has not been investigated.

In this study, we examined lipid metabolism and browning of abdominal and subcutaneous WAT depots in male and female mice during β3-adrenergic stimulation. The selective β3-adrenergic receptor agonist CL316,243 (CL) specifically induced the expression of thermogenic brown adipocyte markers in female gonadal white adipose tissue (gWAT). Interestingly, the level of sympathetic innervation, measured by tyrosine hydroxylase (TH) levels, was significantly greater in gWAT of female mice versus male mice. Levels of nerve growth factor (NGF) and brain-derived neurotrophic factor (BDNF) were significantly greater in gWAT of female versus male mice. Ovarian failure, induced by 4-vinylcyclohexene diepoxide (VCD) treatment, reduced TH protein levels and CL-induced browning of gWAT, similar to the levels observed in male mice, suggesting that differential sympathetic innervation of gWAT is sex hormone dependent. Collectively, these data indicate that differences in sympathetic activity are responsible for the greater ability of females to induce brown adipocytes in gWAT and suggest that the female-specific induction of brown adipocytes in WAT contributes to protection against metabolic disease.

## Methods

### Animals

All animal protocols were approved by the Institutional Animal Care and Use Committees at Yonsei University (A-201605-228-01). All animal experiments were conducted in strict compliance with the guidelines for humane care and use of laboratory animals as specified by the Ministry of Food and Drug Safety. C57BL/6 mice were obtained from Orient Bio (Gyeonggi-Do, South Korea) and were fed a standard chow diet. The mice were housed at 22 °C and maintained on a 12-h light/12-h dark cycle with free access to food and water at all time.

Metabolic measurement was obtained using indirect calorimetry system (PhenoMaster, TSE system, Bad Homburg, Germany). The mice were acclimatized to the cages for 2 days, and O_2_ consumption (VO_2_), CO_2_ production (VCO_2_), food intake and locomotor activity were monitored for 2 days while food and water were provide ad libitum.

For β3-adrenergic receptor stimulation, the mice were injected with CL316,243 (Sigma) (intraperitoneal injection, 1 mg/kg) daily for up to 5 days. The mice were euthanized in the ad libitum fed state after 4 h, 3 days, or 5 days of CL treatment, and WAT and serum were collected. To induce ovarian failure, 4-week-old mice were intraperitoneally injected daily with 4-vinylcyclohexene diepoxide (VCD) (Sigma) at a concentration of 150 mg/kg for 15 consecutive days [20]. The control animals were injected with a sesame oil vehicle control. Vaginal cytology was monitored daily to determine ovarian failure as previously described [20]. Serum concentrations of 17 beta-estradiol were determined by ELISA (Abcam, MA, USA), according to the manufacturer’s instruction.

Mitochondrial electron transport activity of adipose tissue minces were detected in situ by measuring the reduction of 2,3,5-triphenyltetrazolium chloride (TTC, Sigma), as previously described [21]. Alternatively, to measure the O_2_ consumption rate (OCR) of adipose tissue, a piece of minced adipose tissue (~5 mg) were plated in Seahorse XF24 Cell Culture Microplates with XF base medium (Seahorse bioscience), containing 4.5 g/l of glucose, l-glutamine, and sodium bicarbonate and the concentration of O_2_ in media was monitored using Seahorse X24e/XF24 analyzers (Seahorse Bioscience) at 37 °C. XF cell Mito stress test kit (Seahorse Bioscience) was used to measure mitochondrial function with a final concentration of 1 μM oligomycin and 0.5 μM rotenone. Uncoupled respiration was calculated by subtraction of rotenone-induced OCR from oligomycin A-induced OCR [22].

In vivo lipolysis was monitored by serum levels of glycerol and FFA using the free glycerol reagent (Sigma) and FFA detection kit (HR Series NEFA-HR (2), Wako), respectively, according to the manufacturer’s instructions.

### Fractionation of adipocytes and stromovascular cells in WAT and cell cultures

Stromovascular cells (SVC) and adipocyte fractions were isolated from mouse gWAT as previously described [7]. Fractionated adipocytes and SVC were used for messenger RNA (mRNA) analysis. For primary cell culture, PDGFRα + adipocyte progenitors were isolated from SVC from gWAT of control mice by magnetic cell sorting (MACS). PDGFRα^+^ cells were cultured to confluence in growth medium (Dulbecco’s modified Eagle’s medium, DMEM; Sigma) supplemented with 10% fetal bovine serum (FBS; Gibco) and 1% penicillin/streptomycin (Gibco) at 37 °C in a humidified atmosphere with 5% CO_2_ and exposed to differentiation medium (DMEM supplemented with 10% FBS, 1% penicillin/streptomycin, 2.5 mM isobutylmethylxanthine (Cayman Chemical), 1 μM dexamethasone (Cayman Chemical), and 1 μg/ml insulin (Sigma)) for 3 days and maintained in maintenance medium (DMEM supplemented with 10% FBS, 1% penicillin/streptomycin, 1 μg/ml insulin) for 4 days.

### Immunohistochemistry and immunocytochemistry

Adipose tissues were fixed and processed for histological analysis, as previously described [7]. Paraffin sections (5-μm thick) were subjected to immunohistochemical analysis, as previously described [7]. The antibodies used for immunochemical detection were anti-UCP1 antibody (rabbit, 0.5 μg/ml, Alpha Diagnostic International), perilipin 1 (rabbit, 1:100, Cell Signaling), and tyrosine hydroxylase antibody (mouse, 1:400, Merck Millipore). Secondary antibodies used were goat anti-rabbit-Alexa Fluor 488 and goat anti-mouse-Alexa Fluor 594 (1:500, Invitrogen, Molecular Probes). IgG controls (normal rabbit IgG, Santa Cruz) were used as negative controls for IHC analysis, when the information on the concentration of primary antibodies was available (Additional file 1: Figure S1). Otherwise, the omission of primary antibody was used as a negative control. DAPI (Sigma) was used as a nuclear counter stain.

### Gene expression

RNA was extracted using TRIzol® reagent (Invitrogen) and converted into complementary DNA (cDNA) using High Capacity cDNA synthesis kit (Applied Biosystems, Waltham, MA, USA). Quantitative real-time polymerase chain reaction (PCR) was performed using SYBR Green Master Mix (Applied Biosystems) and ABI StepOne PLUS (Applied Biosystems) for 45 cycles, and the fold change for all samples was calculated by the comparative cycle-threshold (Ct) method (i.e., 2−ΔΔCt method). Peptidylprolyl isomerase A (PPIA) was used as the housekeeping gene for mRNA expression analysis. There was no significant difference in Ct values of PPIA among adipose tissue samples from the experimental groups. cDNA was amplified using the following primers for NGF: 5′-ACAGCCACAGACATCAAGGG-3′ (forward), and 5′-TGACGAAGGTGTGAGTCGTG-3′ (reverse). The primers used for BDNF were as follows: 5′-CACTCCACTGCCCATGATGT-3′ (forward), and 5′-GGACCAAAATGGGAGGAGGG-3′ (reverse). All other cDNAs were amplified using previously described primers [7, 23].

### Western blot analysis

Protein extracts were prepared as previously described [13]. Western blotting was performed using primary antibodies against adipose triglyceride lipase (ATGL) (rabbit, Cell Signaling), hormone sensitive lipase (HSL) (rabbit, Cell Signaling), p-HSL (rabbit, Cell Signaling), tyrosine hydroxylase (TH) (mouse, Merck Millipore), UCP1 (rabbit, Alpha Diagnostic International) α/β tubulin (rabbit, Cell Signaling), β-actin (mouse, Santa Cruz Biotechnology), and Total OXPHOS Rodent WB Antibody Cocktail (Abcam) including CI subunit NDUFB8, CII-30 kDa, CIII Core protein 2, CIV subunit I, and CV alpha subunit. Secondary anti-mouse/rabbit horseradish peroxidase antibodies were as described previously [13]. Blots were visualized with SuperSignal West Dura Substrate (Pierce, Invitrogen).

### Statistical analysis

Statistical analyses were performed using GraphPad Prism 5 software (GraphPad Software, La Jolla, CA, USA.). Data are presented as mean ± SEM. Statistical significance between two groups was determined by unpaired *t* test or Mann-Whitney test, as appropriate. Comparison among multiple groups was performed using a one-way analysis of variance (ANOVA) or two-way ANOVA, with Bonferroni post hoc tests to determine *p* values.

## Results

### Browning of WAT by β3-adrenergic receptor stimulation is higher in gWAT of female than male mice

Six-week-old male and female mice were treated with a selective β3-adrenergic receptor agonist, CL for 3 days, and mRNA and protein levels of brown adipocyte markers and mitochondrial function was analyzed in abdominal and subcutaneous WAT. gWAT and inguinal WAT (iWAT) were analyzed as representative dissectible abdominal and subcutaneous WAT, respectively.

**Fig. 1:**
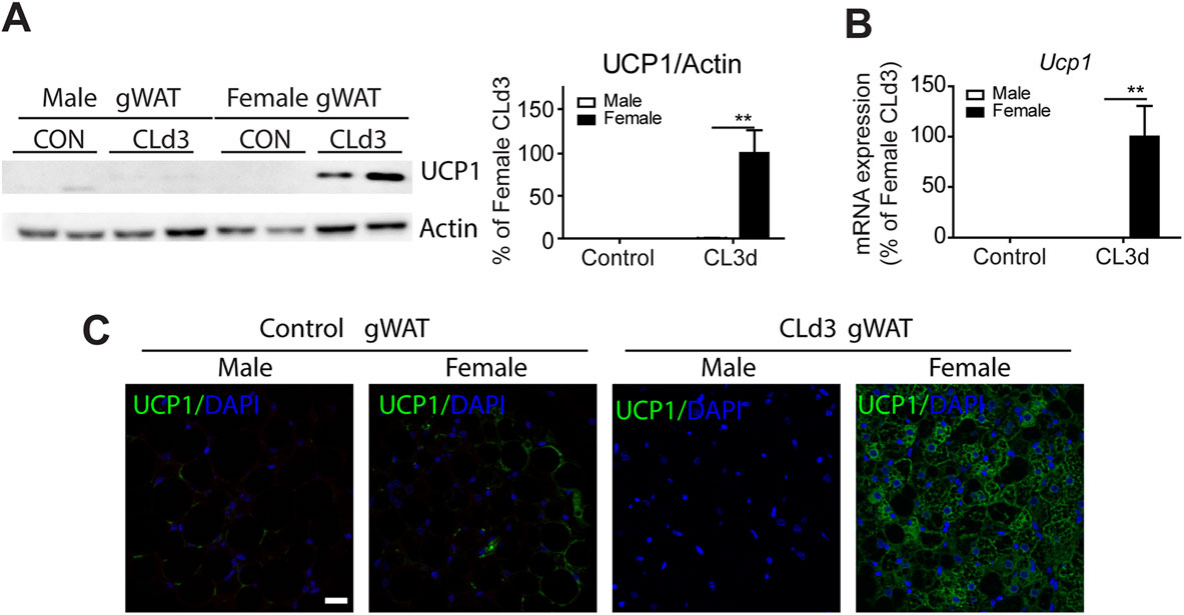
CL treatment induces brown adipocyte maker UCP1 expression in gWAT female specifically. **a**, **b** Immunoblot (**a**) and quantitative PCR (**b**) analysis of UCP1 expression in gWAT of male and female mice treated with CL for 3 days and untreated control mice. Two-way ANOVA revealed significant main effects of sex in UCP1 expression (**a**: *p* = 0.012, **b**: *p* = 0.032) and significant interaction of sex and treatment (**a**: *p* = 0.012, **b**: *p* = 0.033). Significant differences between male and female were determined by post hoc pairwise comparison with Bonferroni correction (*p* = 0.012, mean ± SEM; *n* = 4, **p < 0.01). **c** Representative images of UCP1 immunostaining in paraffin sections of gWAT from male and female mice treated with CL for 3 days and untreated control mice. Nuclei were counterstained with DAPI. *Size bar* = 20 μm

**Fig. 2:**
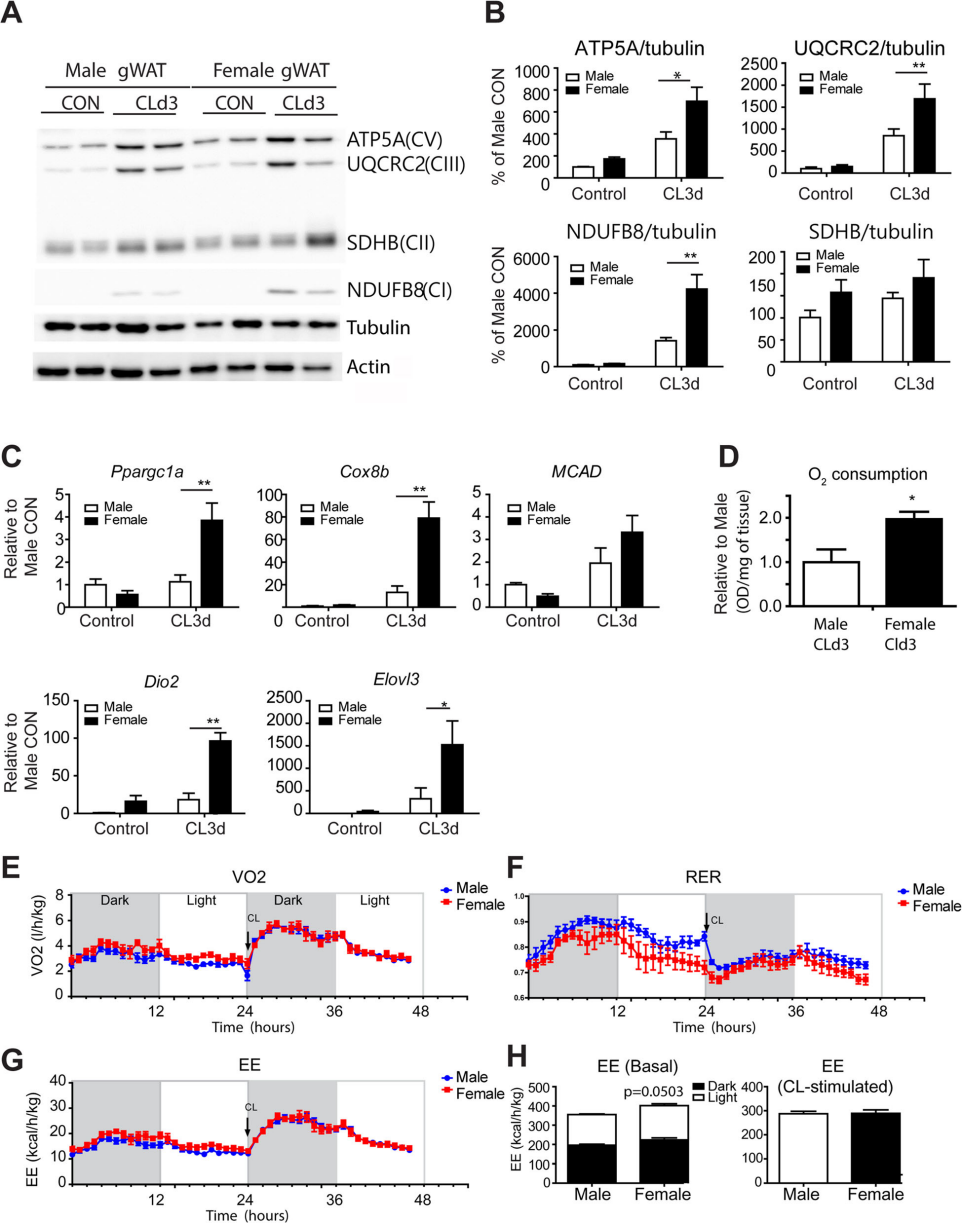
CL treatment induces expression of brown adipocyte markers in gWAT female specifically. **a**,**b** Immunoblot analysis of mitochondrial proteins involved in oxidative phosphorylation. Two-way ANOVA revealed significant main effects of sex in mitochondrial proteins (ATP5A: *p* = 0.013, UQCRC2: *p* = 0.034, NDUFB8: *p* = 0.004) and significant interaction of sex and treatment (NDUFB8: *p* = 0.0054). Significant differences between male and female were determined by post hoc pairwise comparison with Bonferroni correction (mean ± SEM; *n* = 6, *p < 0.05, **p < 0.01). **c** qPCR analysis of brown adipocyte markers and genes involved in mitochondrial FFA oxidation in gWAT of male and female mice treated with CL for 3 days and untreated control mice. Two-way ANOVA revealed significant main effects of sex in brown adipocyte markers (Ppargc1a: *p* = 0.042, Cox8b: *p* = 0.011, Dio2: *p* <0. 0001) and significant interaction of sex and treatment (Ppargc1a: *p* = 0.008, Cox8b: *p* = 0.013, Dio2: *p* = 0. 001). Significant differences between male and female were determined by post hoc pairwise comparison with Bonferroni correction (mean ± SEM; *n* = 4, **p < 0.01). **d** Mitochondrial respiration in gWAT of male and female mice treated with CL for 3 days (mean ± SEM; *n* = 4, *p < 0.05) as determined by reduction of the electron acceptor dye TTC. **e**–**h** VO2 (**e**), energy exchange ratio (RER) (**f**), and energy expenditure (EE) (**g**) are shown. **h** Total EE for 24 h (dark, 12 h; light, 12 h) without stimulation (**h**, *left panel*) and total EE for 12 h after CL injection (**h**, *right panel*). *Arrows* indicate the time of CL injection. Mean ± SEM, *n* = 6 per group

We found that in gWAT, CL treatment robustly induced expression of UCP1 protein in female mice but was without effect in males (Fig. 1a). Consistently, qPCR and immunohistochemical analysis showed that UCP1 expression was greatly upregulated in female but not male mice (Fig. 1b, c). We examined several mitochondrial proteins that constitute the mitochondrial respiratory chain: NDUFB8 (CI subunit: NADH Dehydrogenase [Ubiquinone] 1 Beta Subcomplex 8), SDHB (CII subunit: Succinate dehydrogenase complex iron-sulfur subunit B), UQCRC2 (CIII Core protein 2, Ubiquinol-Cytochrome C Reductase Core Protein II), and ATP5A (CV alpha subunit-ATP synthase, H+ transporting, mitochondrial F1 complex, alpha subunit 1). Of the mitochondrial respiratory chain components analyzed, subunits CV, CIII, and CI were expressed at significantly higher levels in gWAT of female mice treated with CL for 3 days compared to males (Fig. 2a, b). Similarly, functional analysis by TTC staining demonstrated that O_2_ consumption was higher in female gWAT than male gWAT (Fig. 2d). Cytochrome c oxidase subunit VIIIb (Cox8b) and peroxisome proliferator-activated receptor, gamma, and coactivator 1 alpha (Ppargc1a), a transcription factor that upregulates mitochondrial biogenesis were also expressed significantly higher in female gWAT after 3 days of CL treatment (Fig. 2c). Expression of other brown adipocyte markers, deiodinase iodothyronine type II (Dio2), and elongation of very long chain fatty acids 3 (Elovl3) was significantly higher in gWAT of female mice than in gWAT of male mice (Fig. 2c). These results indicate that gWAT of female mice is highly susceptible to browning of WAT upon β3-adrenergic stimulation.

To compare whole-body energy expenditure, we performed indirect calorimetry analysis (Fig. 2e–g). There was no significant sex difference in O_2_ consumption, respiratory exchange ratio (VCO_2_/VO_2_), or energy expenditure. Although there was a significant difference in browning of gWAT, its contribution to whole-body energy expenditure is relatively low compared to that of classic BAT and thus would be difficult to discern by indirect calorimetry.

**Fig. 3:**
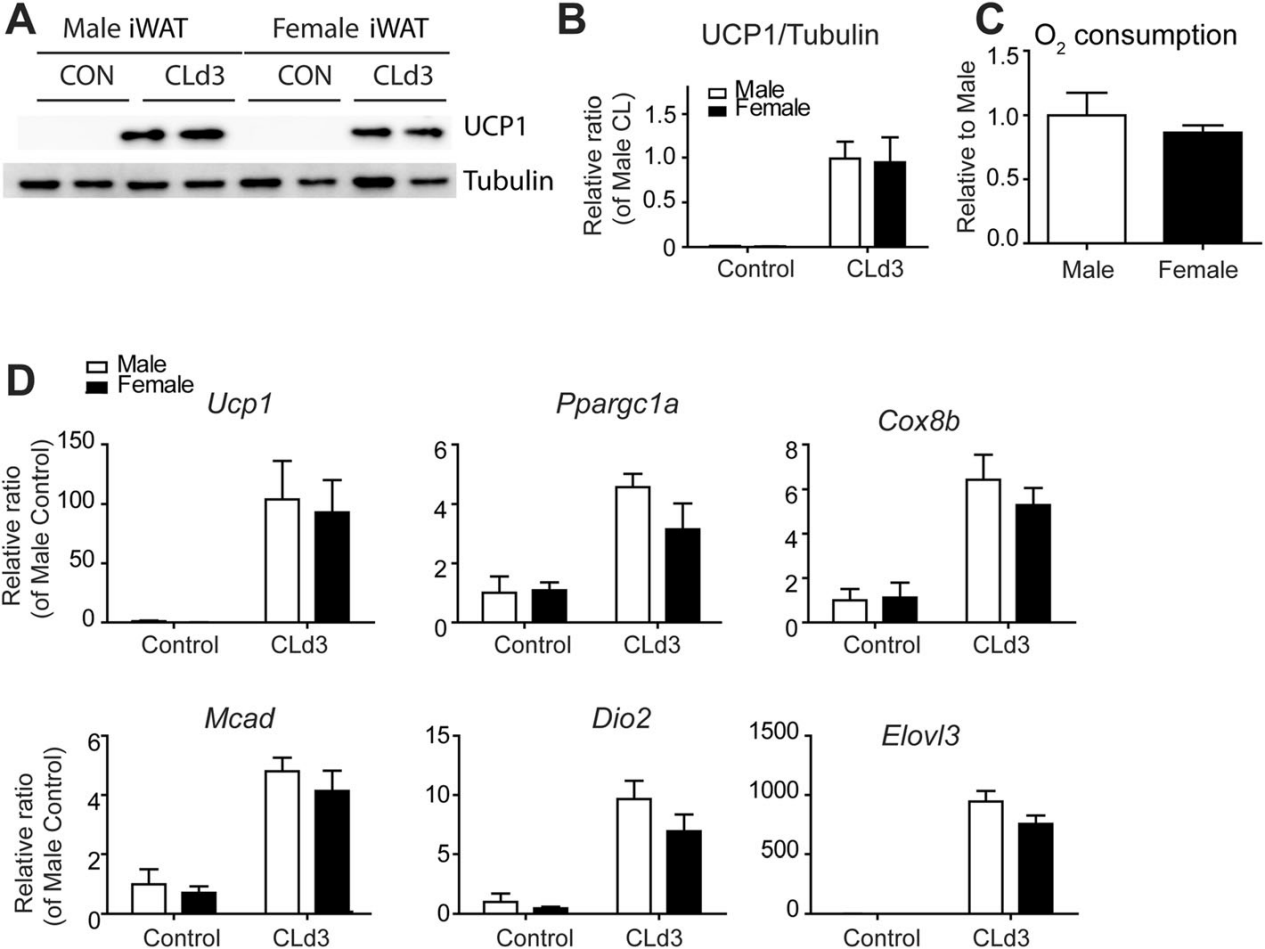
Induction of brown adipocyte phenotype in subcutaneous adipose tissue by CL treatment. **a**, **b** Immunoblot analysis of UCP1 protein expression and quantification. Mean ± SEM; *n* = 4, two-way ANOVA, interaction of sex and treatment (*p* = 0.9094) and effect of sex (*p* = 0.8949) were not significant. Significant main effect of CL treatment (*p* < 0.0001). **c** Mitochondrial respiration in brown adipose tissue and iWAT of male and female mice treated with CL for 3 days (mean ± SEM; *n* = 4) as determined by reduction of the electron acceptor dye TTC. **d** qPCR analysis of brown adipocyte markers and genes involved in mitochondrial FFA oxidation in iWAT of male and female mice treated with CL for 3 days and untreated control mice. (mean ± SEM; *n* = 4)

In contrast to gWAT, CL was equally effective in inducing brown adipocyte markers in inguinal WAT of female and male mice (Fig. 3a, b, d). Levels of mitochondrial respiration measured by TTC staining were also similar between male and female iWAT (Fig. 3c). To determine intrinsic differences in the potential of browning of WAT derived from precursors in female and male adipose tissues, we performed primary cultures with PDGFRα^+^ cells isolated from gWAT and did not find any significant differences in the induction levels of brown adipocyte markers in response to isoproterenol treatment (Additional file 1: Figure S2).

### Lipolysis in response to β3-adrenergic receptor stimulation is higher in gWAT of male than female mice

**Fig. 4:**
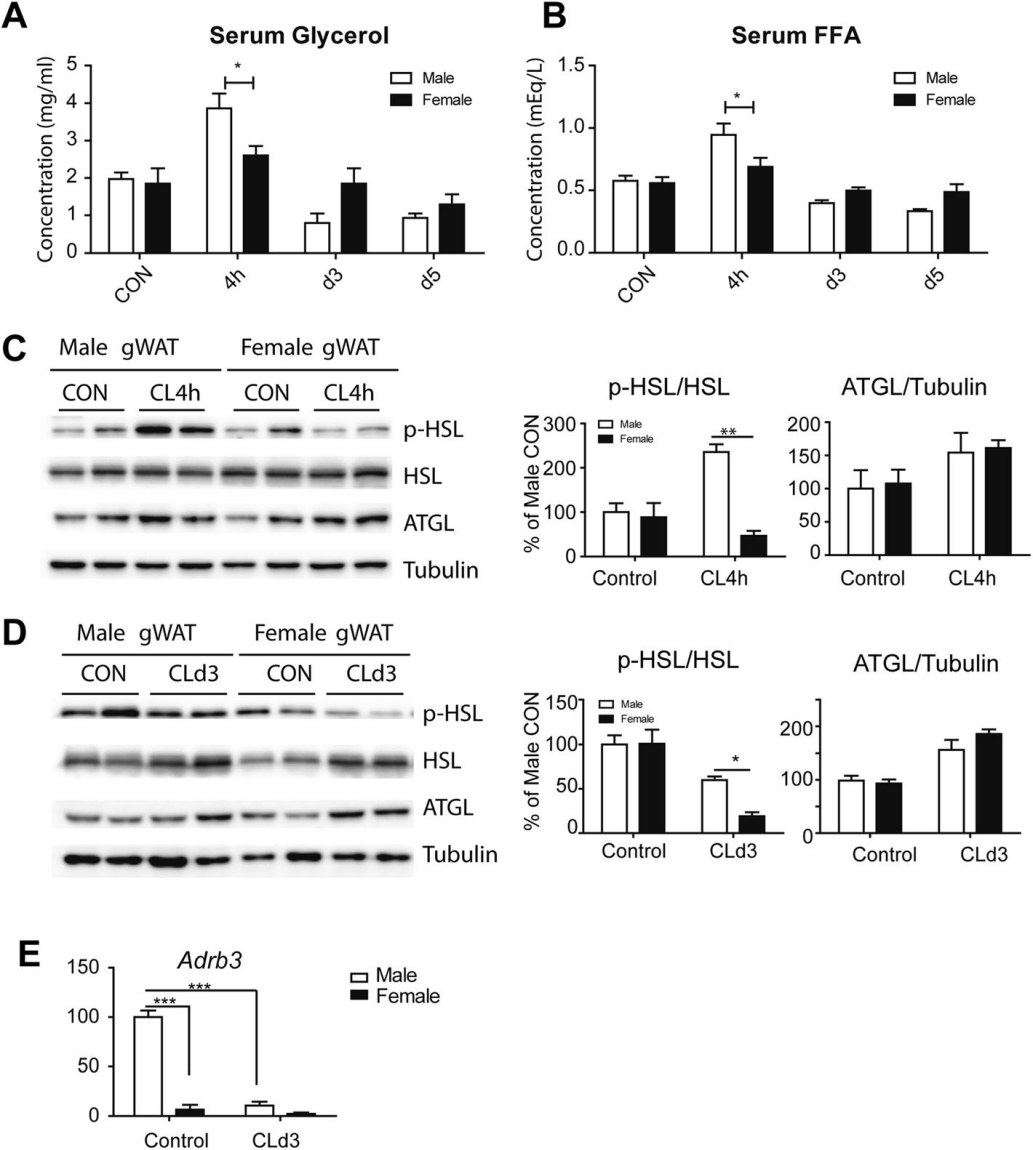
Sex differences in the effects of β3-adrenergic receptor activation on lipolysis. **a**, **b** Sex differences in the effects of β3-adrenergic receptor activation on glycerol (**a**) and free fatty acid levels (**b**) in serum. Mice were treated with CL316,243 up to 5 days, and glycerol and FFA levels in serum were measured. (Mean ± SEM; *n* = 4, *p < 0.05). **c**, **d** Immunoblot analysis of p-HSL, HSL, and ATGL in gWAT of mice treated with CL for 4 h or 3 days and untreated controls. Two-way ANOVA revealed significant main effects of sex in p-HSL (4 h: *p* = 0.014, 4 h: *p* = 0.040). Significant differences between male and female were determined by post hoc pairwise comparison with Bonferroni correction (mean ± SEM; *n* = 4, *p<0.05, **p < 0.01). **e** qPCR analysis of Adrb3 expression in gWAT of mice treated with CL for 3 days and untreated controls. Two-way ANOVA revealed significant main effects of sex in Adrb3 expression (*p* < 0.001) and significant interaction of sex and treatment (*p* < 0.001). Significant differences between male and female were determined by post hoc pairwise comparison with Bonferroni correction (mean ± SEM; *n* = 4, ***p < 0.001)

We next addressed the mechanisms involved in the sex differences in UCP1 induction. We examined the acute lipolytic responsiveness of male and female mice to CL because FFA are known PPAR ligands that support catabolic remodeling of gWAT [24]. Surprisingly, male mice were more responsive, indicated by greater elevation of serum FFA and glycerol after 4 h of CL treatment (Fig. 4a, b). Hormone sensitive lipase (HSL) and adipose triglyceride lipase (ATGL) are the major enzymes responsible for triglyceride hydrolysis in adipose tissue. Therefore, we examined the expression levels of HSL and ATGL Immunoblot analysis showed that CL sharply elevate phosphorylation of HSL in male but not in female mice (Fig. 4c). These observations indicate that the acute intrinsic responsiveness gWAT to CL is not diminished in male mice. Following treatment with CL for 3 days, the levels of phosphorylated HSL returned to basal levels (Fig. 4d). In contrast, there were no sex differences in the basal levels and CL-induced upregulation of HSL and p-HSL levels in iWAT in response to CL treatment (Additional file 1: Figure S3).

Interestingly, Adrb3 expression was higher in gWAT of male mice compared to female mice and was sharply downregulated by 3 days of CL treatment (Fig. 4e). This difference in the expression of Adrb3 in gWAT between sexes may explain why β3-adrenoceptor stimulation acutely increased the activation of the lipolysis pathway in male gWAT.

### Sympathetic innervation levels are significantly higher in gWAT of female mice than male

**Fig. 5:**
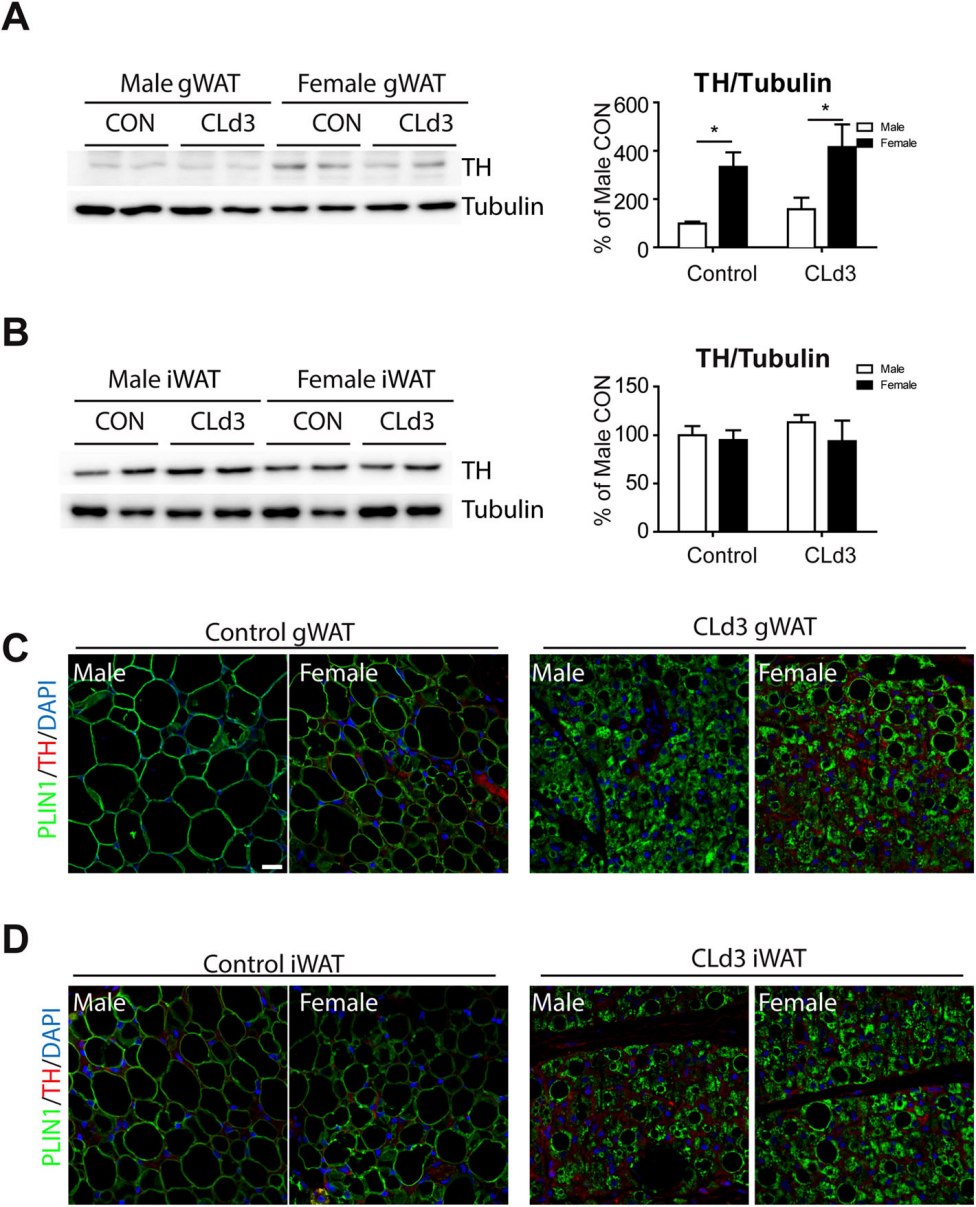
Sympathetic innervation levels in gWAT and iWAT of male and female mice. **a**, **b** Immunoblot analysis of tyrosine hydroxylase (TH) protein expression in gWAT and iWAT of mice treated with CL for 3 days and untreated controls. Two-way ANOVA revealed significant main effects of sex in TH protein expression (*p* = 0.0006). Significant differences between male and female were determined by post hoc pairwise comparison with Bonferroni correction (mean ± SEM; *n* = 4, *p < 0.05) (**c**, **d**). Representative images of TH and PLIN1 staining in paraffin sections of gWAT and iWAT from mice treated with CL for 3 days and untreated controls. Nuclei were counterstained with DAPI. *Size bar* = 20 μm

It is well established that the metabolic activity of brown adipose tissue is controlled by sympathetic nerve activity [5]. In addition to metabolic activation, the potential of WAT to induce brown adipocyte phenotypes is proportional to the basal levels of sympathetic innervation [12]. Therefore, we hypothesized that differential levels of sympathetic innervation might affect recruitment of brown adipocyte phenotypes in gWAT of each sex. To measure the level of sympathetic innervation, we performed immunoblot analysis of the TH protein, the enzyme that mediates the rate-limiting step of norepinephrine biosynthesis. We found the TH levels were threefold higher in gWAT of females versus male gWAT (Fig. 5a). Consistent with this result, immunohistochemical analysis revealed that gWAT of female mice treated with CL contains more TH+ nerve fibers (Fig. 5c). In contrast to gWAT, there were no sex differences in TH protein levels, indicated by immunoblot and IHC analysis (Fig. 5b, d).

### Neurotrophic factors are significantly higher in gWAT of female mice than male

**Fig. 6:**
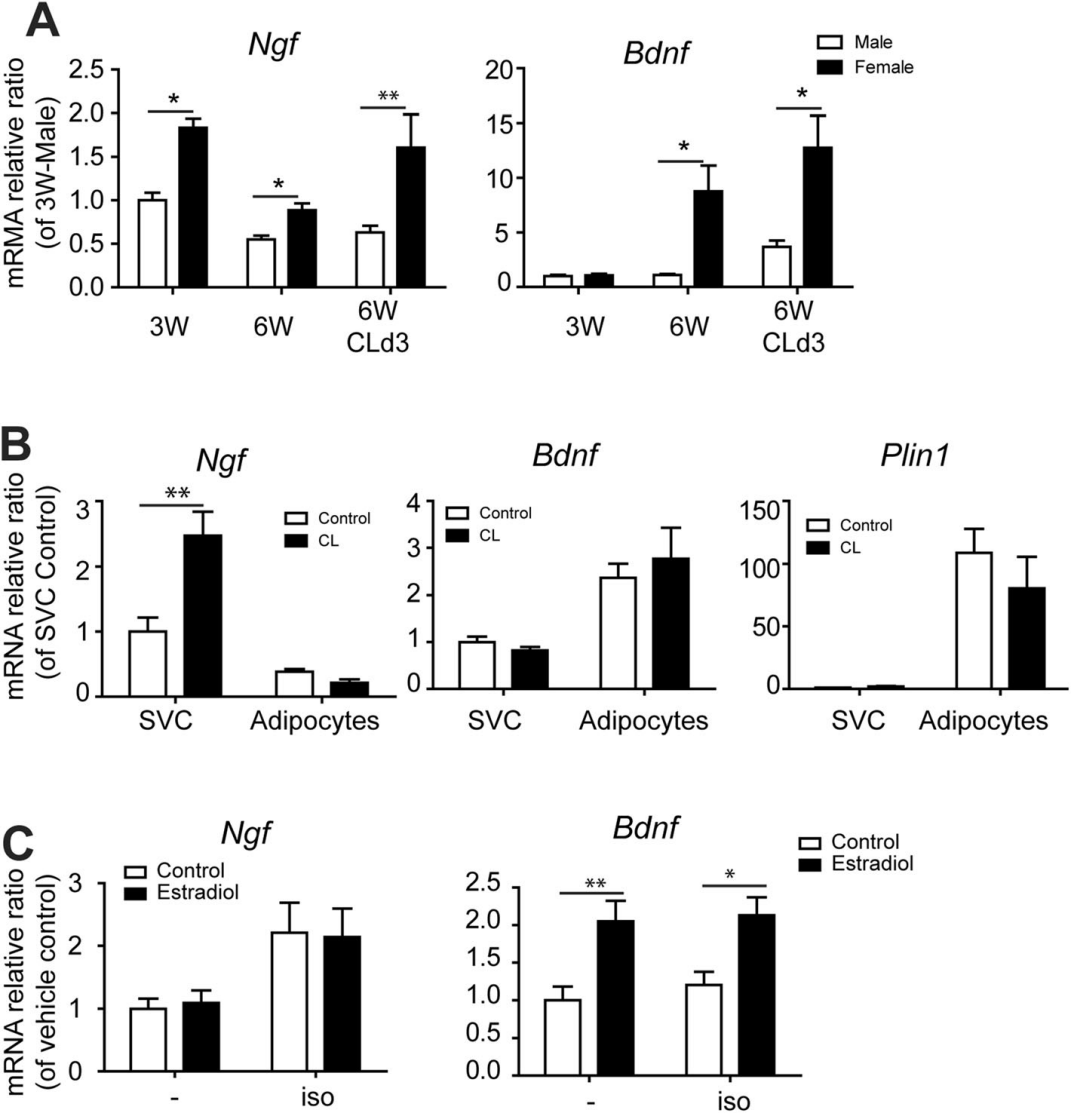
Sex differences in NGF and BDNF expression in gWAT of weanling mice and during β3-adrenergic receptor activation. **a** qPCR analysis of NGF and BDNF expression in gWAT of weanling mice (3 weeks of age), 6-week-old mice treated with CL for 3 days (6W-CL), and untreated control mice (6W). **b** qPCR analysis of NGF and BDNF expression in SVC and adipocytes obtained from gWAT of female mice treated with CL for 3 days and untreated controls. **c** qPCR analysis of NGF and BDNF expression in differentiated adipocyte from PDGFRα progenitors obtained from gWAT of mice. Primary cultured adipocytes were treated with 17β-estradiol (10 nM) or vehicle in the presence or absence of 10 μM isoproterenol for 1 day (mean ± SEM; *n* = 4, *p < 0.05, **p < 0.01)

To identify potential factors that affect sympathetic innervation, we examined levels of neurotrophic factors [25]. Interestingly, NGF expression was slightly higher in gWAT of female mice and was significantly upregulated by CL treatment (Fig. 6a). BDNF expression levels were also higher in gWAT of female mice. However, BDNF expression in gWAT of female mice was not upregulated following CL treatment (Fig. 6a). Because it has been reported that NGF regulates axonal outgrowth and the developmental targeting of postganglionic sympathetic nerves to target tissues [25], we examined developing adipose tissues from weanling mice. We found that NGF levels in gWAT were twofold higher in weanling females compared to males. The NGF levels declined by 6 weeks of age, and NGF could be upregulated by 3 days of CL treatment in female, but not male, mice (Fig. 6a).

Adipose tissue is a mixture of cell types. To determine which cell types express neurotrophic factors, gWAT was fractionated into stromovascular cells (SVC) and adipocytes. While the expression levels of NGF were slightly higher in SVC compared to levels in adipocytes under control conditions, CL treatment significantly increased NGF expression in SVC, but not in adipocytes (Fig. 6b). BDNF expression was 2.5-fold higher in adipocytes fraction compared to SVC, and the expression was not affected by CL treatment (Fig. 6b).

To determine sex hormone effects on beige/BRITE adipocyte characteristics, primary cultured adipocytes from WAT of mice were treated with 17β-estradiol. In line with CL effect in vivo, β-adrenergic receptor agonist, isoproterenol increased NGF expression, but not BDNF. In addition, we found that estradiol treatment increased BDNF levels (Fig. 6c), suggesting that estradiol increases the production of neurotrophic factors in adipocytes, resulting in higher levels of innervation in female gWAT.

### Sex hormone is required for beige/BRITE adipose phenotype of gonadal adipose tissue

**Fig. 7:**
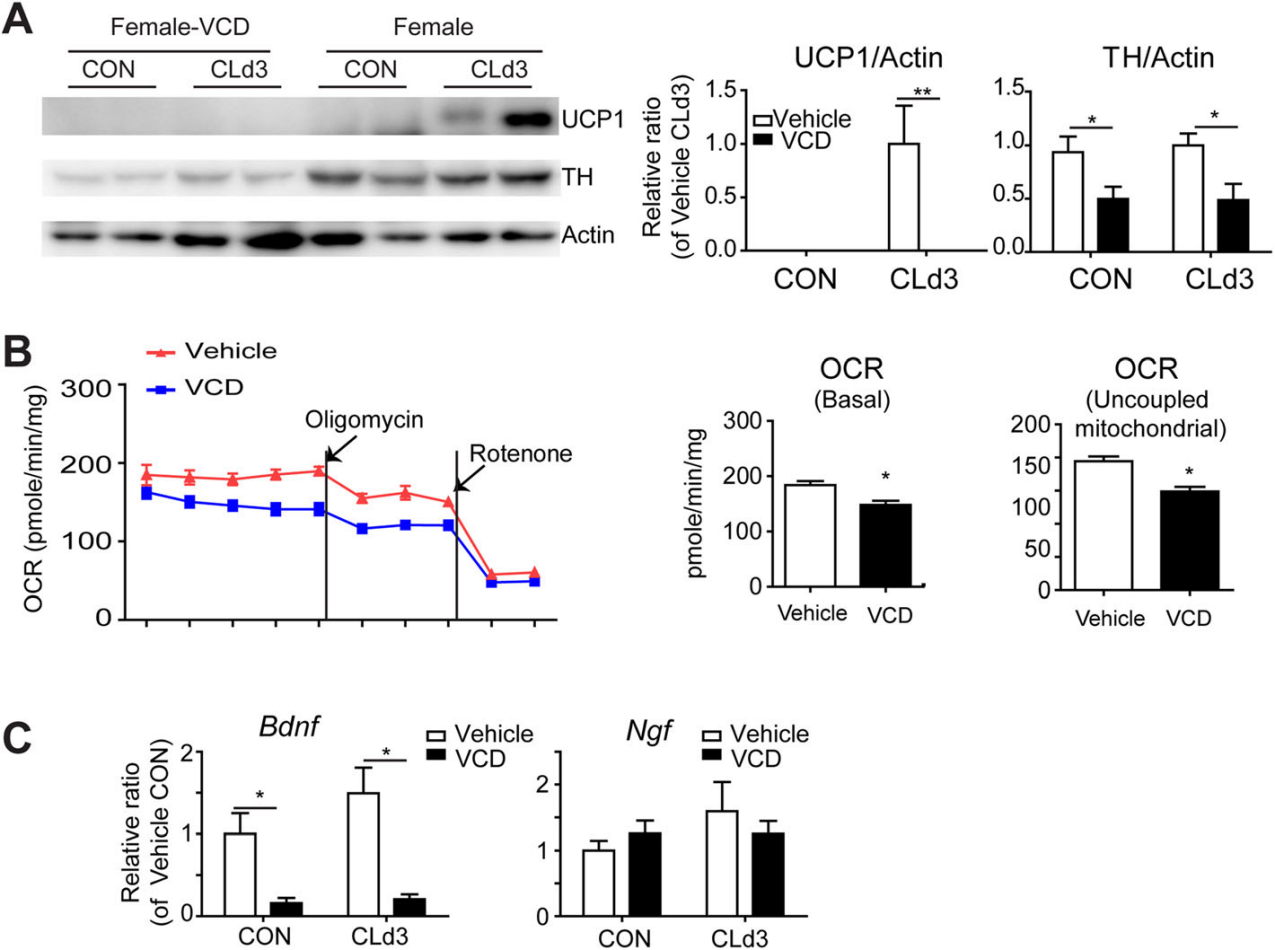
4-Vinylcyclohexene diepoxide induced ovarian failure reduced TH levels and CL-induced browning of gWAT of female mice. **a** Immunoblot analysis of UCP1 and TH protein in gWAT of vehicle- and VCD-treated mice after 3 days of CL treatment and control mice. Two-way ANOVA revealed significant main effects of VCD treatment in TH (*p* = 0.0037) and UCP1 expression (*p* = 0.015). Significant differences between controls and VCD-treated female mice were determined by post hoc pairwise comparison with Bonferroni correction (mean ± SEM; *n* = 4, *p < 0.05, **p < 0.01) (**b**). Basal and oligomycin- and rotenone-induced oxygen consumption rate of gWAT obtained from vehicle- and VCD-treated mice treated with CL for 3 days. **c**. qPCR analysis of NGF and BDNF expression in gWAT of vehicle- and VCD-treated mice after 3 days of CL treatment, and control mice. Two-way ANOVA revealed significant main effects of VCD treatment in BDNF expression (*p* = 0.0079). Significant differences between controls and VCD-treated female mice were determined by post hoc pairwise comparison with Bonferroni correction (mean ± SEM; *n* = 4, *p < 0.05)

To determine whether sex hormones are required for the beige/BRITE adipose phenotype of gonadal adipose tissue, we used the 4-vinylcyclohexene diepoxide (VCD) model to induce ovarian failure and thereby remove the source of estrogen [20]. Controls and mice with chemically induced ovarian failure were treated with CL, and TH and UCP1 protein levels were determined by immunoblot analysis. VCD treatment decreased innervation levels and abolished the ability of CL to induce UCP1 expression (Fig. 7a). Real-time metabolic analysis showed that basal and uncoupled mitochondrial respiration were reduced in gWAT of VCD-treated mice compared to vehicle treated mice after 3 days of CL treatment (Fig. 7b). In addition, levels of BDNF mRNA expression were significantly reduced in gWAT of VCD-treated mice compared to vehicle treated mice (Fig. 7c).

## Discussion

Obesity increases cardiometabolic risk in males, yet the correlation in females is less clear [2]. Furthermore, epidemiologic studies and in vivo experiments support the observation that females have lower cardiometabolic risk compared to males with similar levels of adiposity [2, 26]. Sex hormones influence body adiposity as well as the regional distribution of adipose tissue [18]. Therefore, it is possible that the sex dimorphisms observed in the pathophysiology of metabolic disease are associated with sex-difference in the metabolic function of adipose tissue. In general, increasing in the mass of subcutaneous adipose tissue is beneficial to metabolic profiles, while abdominal adipose tissue is related to insulin resistance and disease states [27]. Thus, higher levels of subcutaneous fat in women have been considered a main factor contributing to female-specific resistance to metabolic disease [2, 26]. However, sex-differences in sympathetic innervation levels and the induction of thermogenic adipocytes in anatomically analogous abdominal WAT have not been investigated. In this study, we hypothesized that the lipid metabolism of anatomically similar abdominal WAT can be affected by sex hormones. To test this, the metabolic phenotypes of anatomically corresponding WAT from male and female mice were analyzed to determine differences between sexes, focusing on the browning of gWAT and iWAT in response to β3-adrenergic stimulation.

Browning of WAT is a promising pathway to increase energy expenditure as well as a potential therapeutic target to combat obesity and related metabolic disease. In this study, we demonstrated that the levels of lipolysis and browning of gWAT differed by sex and this difference is, in part, due to differential levels of sympathetic innervation to gWAT between the sexes. Previous work has shown that tonic sympathetic activity is important in maintaining the ability of WAT to undergo browning in response to CL treatment [12]. Although there is variation between strains of mice, in general, gWAT is considered the most refractory to thermogenic stimuli in male C57BL/6 mice [28, 29]. Our current study shows that gWAT of female C57BL/6 mice was able to adopt a beige/BRITE phenotype. Although the mechanisms are not fully certain, we demonstrated that higher BDNF expression in gWAT of females is sex hormone dependent. The difference in NGF expression between the sexes was greater in the developing gWAT of mice, indicating that NGF may play an important role in the differential innervation of postganglionic sympathetic nervous system to gWAT developmentally. Interestingly, NGF expression was induced by CL treatment, suggesting positive feedback regulation of β-adrenergic tone.

Although BDNF expression is much higher in gWAT of adult female mice (e.g., 6 weeks old), BDNF expression levels did not exhibit any sex differences in developing adipose tissue. Importantly, BDNF expression was upregulated by estrogen treatment in vitro, and VCD-induced ovarian failure reduced BDNF expression. These data indicated that BDNF is an estrogen-sensitive neurotrophic factor that contributes to differential sympathetic innervation of gWAT. Although the mechanism of BDNF induction by estrogen is not determined in this study, the promoter of the BDNF gene contains estrogen response elements (ERE) [30–32]. VCD, a well-established ovarian toxicant, has been used to induce ovarian failure [20]. However, we do not exclude unknown off-target effects of VCD treatment. Thus, further confirmation with surgical ovariectomy models in combination with estrogen replacement would be informative to support ovarian steroid hormone-specific effects on browning of gWAT. As mentioned above, innervation levels in iWAT did not differ between male and female mice, suggesting that distinct mechanisms may be involved in development and maintenance of postganglionic sympathetic neurons in various anatomic locations.

While sex differences in the browning of gWAT have not been previously investigated, the higher metabolic activity of classic brown adipose tissue in females has been previously reported [33–35]. For example, studies using ^18^F-FDG positron emission tomographic and computed tomographic scans indicated that metabolically active BAT is more frequently observed in woman than in men [35]. Previous studies have identified higher levels of BMP8 expression as a molecular mechanism of estrogen-induced upregulation of metabolic activity in classic BAT of female mice [36]. Interestingly, recent studies demonstrated that thermogenic/browning effect of central BMP8b and AMPK activation in hypothalamus is restricted to female, showing estrogen dependency [34, 37, 38]. Further studies are needed to address whether the central regulation is involved in sex-dimorphic browning of gWAT. In addition to controlling BAT metabolism, important roles of estrogen in energy homeostasis have been intensively studied [16, 39]. For example, ovariectomy in rodents impairs glucose tolerance and increases WAT mass [16]. Moreover, studies using knockout mice have shown that estrogen receptor-α suppresses adipose tissue expansion in male and female mice [19].

In addition to sexual difference in browning of gWAT, male and female mice had different lipolytic responses. Generally, both lipolysis and thermogenic response are regulated by sympathetic activity. The current study suggested that greater TH levels maintain the ability to respond to CL for the induction of thermogenic gene expression. However, it is not clear how higher levels of innervation preferentially activated oxidative mechanisms over lipolysis. We speculate that higher levels of innervation might downregulate Adrb3 expression in females, which explain lower lipolytic responsiveness to acute CL treatment [12]. Differential compartments of cAMP-dependent signaling are required for the enzymatic activation of TG hydrolysis and transcriptional activation of the thermogenic program [40, 41]. Thus, it is possible that higher basal levels of sympathetic activity may lead compartmentalization of PKA signaling to nucleus targeted downstream to sensitize thermogenic stimuli. Although levels of phosphorylated HSL in gWAT and serum levels of FFA and glycerol indicate activation of lipolysis in gWAT, we did not monitor lipolytic flux directly. Nonetheless, the sexually dimorphic upregulation of mitochondria in gWAT and elevated oxygen consumption measured ex vivo are consistent with greater oxidation of mobilized FFA in female gWAT.

## Conclusions

We have demonstrated that the sex differences in sympathetic activity result in gWAT beige/BRITE phenotype in female mice and suggest that the distinctively female-specific induction of brown adipocytes in gWAT could be involved in the protection of female mice against metabolic disease. Obesity-related metabolic disease is known as a sex-biased disease. An understanding of sex dimorphism in the physiology and mechanisms of adipose tissue function may lead to the development of new therapeutics to prevent obesity-related metabolic disease.

## Additional file

Additional file 1: Figure S1. Negative controls used for UCP1 immunostaining in Fig. 1c. **Figure S2.** The induction of brown adipocyte markers in adipocytes derived from gWAT of male and female mice. **Figure S3.** Activation of TG hydrolase in iWAT during β3-adrenergic receptor activation.

## Abbreviations

CL: CL316,243
FFA: Free fatty acids
gWAT: Gonadal white adipose tissue
iWAT: Inguinal white adipose tissue
SVC: Stromovascular cells
TH: Tyrosine hydroxylase
TTC: 2,3,5-Triphenyltetrazolium chloride
VCD: 4-Vinylcyclohexene diepoxide
