# Supplementary material for: Sex differences in sympathetic innervation and browning of white adipose tissue of mice
Source: Biol Sex Differ. 2016 Dec 9;7:67. doi: 10.1186/s13293-016-0121-7 (PMC5148917; doi:10.1186/s13293-016-0121-7)
Supplement: Additional file 1: Figure S1. — Negative controls used for UCP1 immunostaining in Fig. 1c. Figure S2. The induction of brown adipocyte markers in adipocytes derived from gWAT of male and female mice. Figure S3. Activation of TG hydrolase in iWAT during β3-adrenergic receptor activation. [file 13293_2016_121_MOESM1_ESM.docx]

**Supplemental figures**


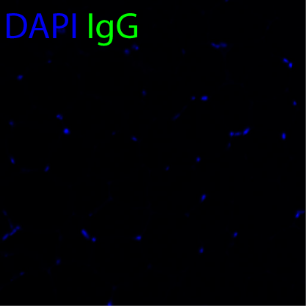


**Figure S1. Negative controls used for UCP1 immunostaining in Figure 1C.**

Normal rabbit IgG controls were used for negative controls for UCP1 immunostaining in Figure 1C. Nuclei counterstained with DAPI.


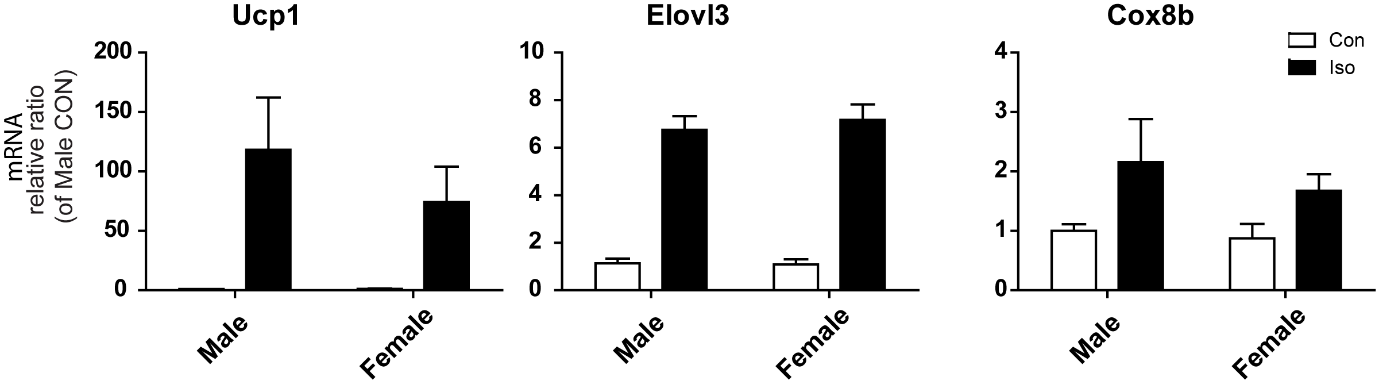


**Figure S2. No differences in the induction of brown adipocyte markers in adipocytes derived from gWAT of male and female mice**.

qPCR analysis of brown adipocyte markers in differentiated adipocytes from PDGFRα+ cells obtained from gWAT of male and female mice. Primary cultured adipocytes were treated with 10 μM isoproterenol (Iso) or vehicle for 1 day For adrenergic stimulation, isoproterenol was used. (mean ± SEM; n=4)


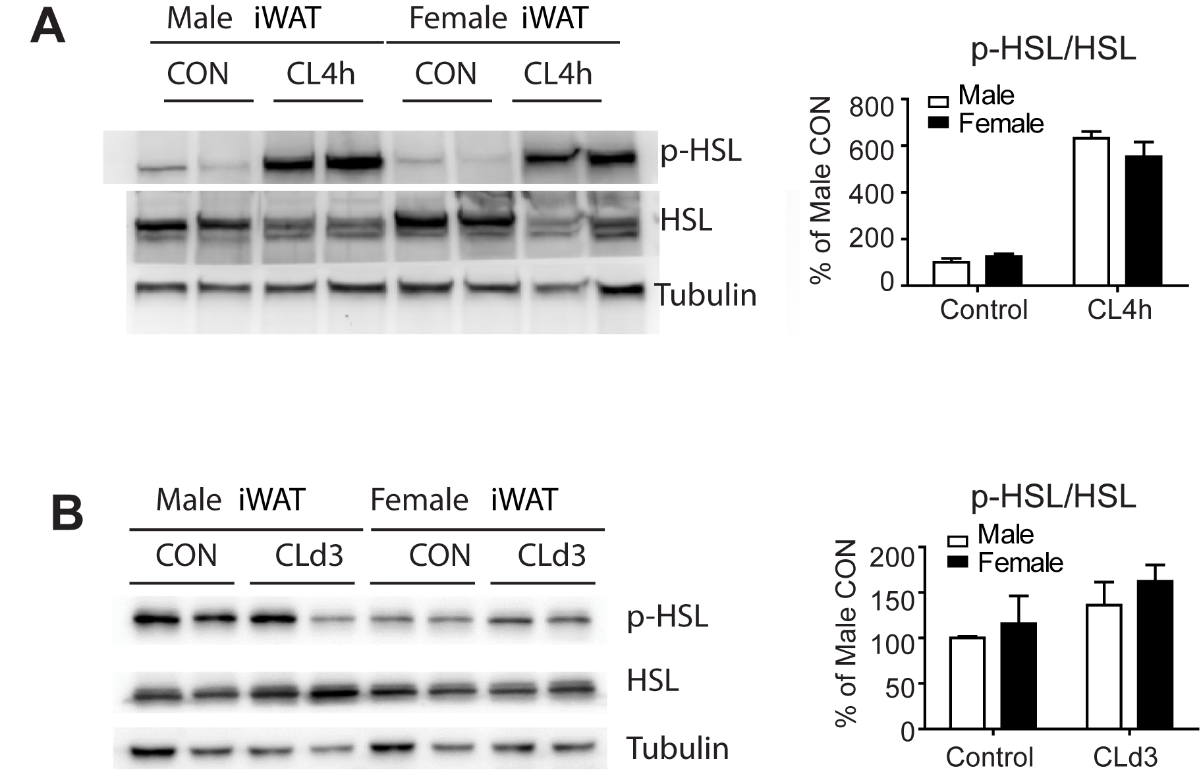


**Figure S3. Activation of TG hydrolase in iWAT during β3-adrenergic receptor activation. I**mmunoblot analysis of HSL and p-HSL in iWAT of mice treated with CL for 4h or 3 days and untreated controls. (mean ± SEM; n=4)


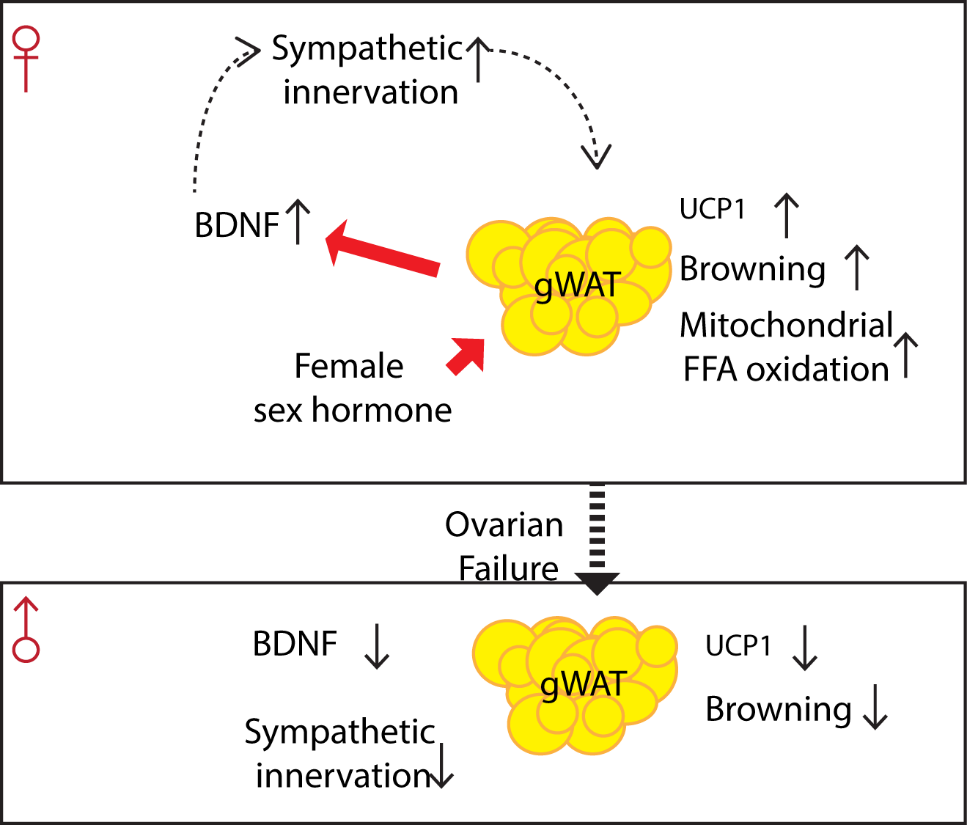


**Figure S4. Graphical abstract: sex-difference in browning of white adipose tissue of mice.**
